# Single-block pulse-on electro-optic Q-switch made of LiNbO_**3**_

**DOI:** 10.1038/s41598-017-05009-5

**Published:** 2017-07-05

**Authors:** Jifang Shang, Jun Sun, Qinglian Li, Jinfeng Yang, Ling Zhang, Jingjun Xu

**Affiliations:** 1 School of Physics, Nankai University, Tianjin, 300071 China; 2 MOE Key Laboratory of Weak-Light Nonlinear Photonics, Nankai University, Tianjin, 300457 China

## Abstract

A novel LiNbO_3_ (lithium niobate, LN) electro-optic (EO) Q-switch that can independently operate in the pulse-on regime without the assistance of a quarter-wave plate (QWP) or analyzer was designed and demonstrated. By theoretical analysis and calculations, the proper orientation of the LN was determined to be θ = 1.7° and φ = ±45°, and the quarter-wave voltage was identical to that of a conventional LN EO Q-switch. Additionally, the possible influences caused by the small angular variation between the wave normal and optic axis were found to be negligible. To the best of our knowledge, this is the first time that a LN crystal has been (xztw)-1.2°/1.2°-cut and used successfully in a pulse-on cavity without using a QWP or analyzer. The performance of the novel Q-switched laser and its temperature dependence were verified to be almost identical to those of a conventional pulse-on LN EO Q-switched laser, which strongly demonstrates the practicability of our novel Q-switch. This novel Q-switch design enables a more compact, lossless and stable laser cavity, which is of great concern for engineering applications.

## Introduction

Lasers with narrow pulse width and high peak power have attracted considerable attention because of their extensive applications in the fields of remote sensing, laser machining, environmental monitoring, medicine, etc.^1, 2^. A common way to realize such pulse sources is to Q-switch a solid-state oscillator. Electro-optic (EO) Q-switching, which is used as an active Q-switching technique, has widely attracted attention^3–5^ due to its advantages over other Q-switching techniques^6^, including its better hold-off ability^7^, controllable repetition rates^8^ and faster switching rate^9^, the latter of which is helpful for achieving a narrow pulse width.

Until now, EO crystals have mainly included KD_2_PO_4_ (DKDP), LiNbO_3_ (LN), β-BaB_2_O_4_ (BBO), KTiOPO_4_ (KTP), RbTiOPO_4_ (RTP), La_3_Ga_5_SiO_14_ (LGS), etc. Among these crystals, LN and DKDP are the two primary EO crystals that have been practical^10^. Recently, other EO crystals, such as BBO, RTP and LGS, have been significantly investigated^11–14^. BBO crystals possess a high optical damage threshold^12^, but their small EO coefficients and short obtainable size along the optic axis are disadvantages for commercial applications^15^. RTP and LGS crystals are favorable for the application of high-repetition-rate EO Q-switching because of their large EO coefficients, high optical damage threshold, insolubility in air and absence of piezoelectric ringing^3^, and remarkable results have been achieved. For example, the repetition rate of a RTP Q-switched laser was reported to be as high as 280 kHz^7^. A recently reported LGS Q-switched laser operated at a repetition rate of 200 kHz^9^. However, some intrinsic problems limit their further engineering applications, such the low-symmetry structure and natural birefringence of RTP^3^ and the optical activity of LGS^16^.

DKDP crystals can be easily grown with a high optical homogeneity^17^, which can satisfy the requirement of a large caliber EO Q-switch. However, DKDP is water soluble and must be carefully protected from moisture, which complicates its fabrication and application^3^. Additionally, the refractive index-matching fluid will disable DKDP EO Q-switches at low temperature. However, a well-known requirement for EO Q-switches is to operate in a wide temperature range, especially for military applications^18^. Fortunately, LN crystals are nonhygroscopic, and they possess a low absorption coefficient and insert loss^19^. In addition, they can operate stably in a wide temperature range^20^, which makes LN crystals the main EO crystal applied in military applications.

Previous reports on LN EO Q-switches have all adopted the configuration wherein an electric field is applied to the X or Y direction, and the light propagates parallel to the optic axis^17^. These Q-switches mainly operate in two modes: pulse-off and pulse-on. In the pulse-off regime, no quarter-wave plate (QWP) or analyzer is needed, thus enabling a compact and stable resonant cavity. Moreover, switching voltage to zero is easier than from zero, and the switching voltage required in the pulse-off regime is usually lower than in the pulse-on regime. This is mainly caused by two factors. First, the constant stress (unclamped) EO coefficient γ^T^ involved in the pulse-off regime is greater than the constant strain (clamped) EO coefficient γ^S^ involved in the pulse-on regime^17^. Second, the full quarter-wave voltage may not always be required to achieve the hold-off state in the pulse-off regime^21^. As a result, the pulse-off regime has been widely studied and applied in industrial applications. Unfortunately, continuously applying a high voltage shortens the lifetime of a Q-switch because of electro-chemical degradation effects^17^. Moreover, in some high-gain lasers, a negative voltage instead of a zero voltage is essential to compensate for the phase retardation induced by the piezoelectric and elasto-optic effects^22^, which is a major requirement for the EO Q-switch driver. The pulse-on regime can compensate for the shortcomings of the pulse-off regime, but then a QWP or analyzer is necessary in the resonant cavity, and using additional optical components is disadvantageous for the compactness and stability of the cavity. Additionally, the wave plate is well known to be very sensitive to temperature and stress^23^, which increases the difficulty of clamping and debugging.

In this work, by integrating the advantages of these two operating modes, we design a novel LN EO Q-switch that can operate in the pulse-on regime without using a QWP or analyzer. The design exploits the idea that LN crystals can be used as a QWP by taking advantage of natural birefringence. The phase retardation and the angle of the eigen polarization direction when an electric field is applied to the X direction and when light propagated near the optic axis are analyzed and calculated. By applying the EO Q-switching theory to these results, the proper orientation of the LN is determined. The results show that θ = 1.7° and φ = ±45°. Additionally, the quarter-wave voltage is found to be equal to that of a conventional LN EO Q-switch. Based on our theoretical analysis, we prepare a single-block pulse-on LN EO Q-switch that is (xztw)-1.2°/1.2°-cut. Its performances and temperature stability were investigated experimentally in a Nd:YAG laser and were found to be almost identical to those of a conventional pulse-on EO Q-switch. This work will be beneficial for engineering applications because the novel pulse-on Q-switch is more conducive to the compactness and stability of the cavity than the conventional one, while retaining the Q-switching performance and a long operational lifetime.

## Methods

### Theoretical analysis

To provide a theoretical basis, the phase retardation and the eigen polarization direction for light propagating near the optic axis when an electric field is applied or not applied are first analyzed. For a LN crystal which is uniaxial, the phase retardation induced by natural birefringence for light propagating along an arbitrary direction can be expressed as^24^

1 $${\delta }_{1}=\frac{\pi l\Delta {{n}_{o}}^{3}{\sin }^{2}\theta }{\lambda }$$

where $\Delta =\frac{1}{{n}_{e}^{2}}-\frac{1}{{n}_{o}^{2}}$, *θ* is the angle between the optic axis and the wave normal, *l* represents the optical path length, and *n*_*o*_ and *n*_*e*_ are the o- and e- refraction indexes in the LN crystal, respectively. One eigen polarization direction of the wave in the crystal is in the principal plane, and the other is perpendicular to this plane.

When an electric field E is applied to the X direction of LN, the index ellipsoid will change due to the EO effect^25^. The birefringence and the angle of eigen polarization direction can be obtained using the expression given by Mason for calculating the birefringence in any direction in a crystal of lowest symmetry^26^. In our design, only small values of *θ* are considered, for which cos *θ* ≈ 1 and sin *θ* ≈ *θ*. Moreover, the terms induced by the electric field are far less than the terms that contribute to the natural birefringence in the optical impermeability tensor. Thus, the phase retardation induced by the electric field can be expressed as

2 $${\delta }_{2}=\frac{\pi {{n}_{o}}^{3}l}{\lambda }{[{(\Delta {\sin }^{2}\theta -2{\gamma }_{22}E)}^{2}+4{\gamma }_{22}E\Delta {\sin }^{2}\theta (1-\sin 2\phi )]}^{1/2}$$

where the high-order terms have been neglected. A similar simplification can be found in the literature^27^. In the equation, *γ*_22_ is the EO coefficient, and *φ* represents the azimuthal angle of the wave normal. The angle *ψ* of the eigen polarization direction to the principal plane can be derived as

3 $$\tan \,2\psi =\frac{2{\gamma }_{22}E\,\cos \,2\phi }{2{\gamma }_{22}E\,\sin \,2\phi -\Delta {\sin }^{2}\theta }$$

**Figure 1:**
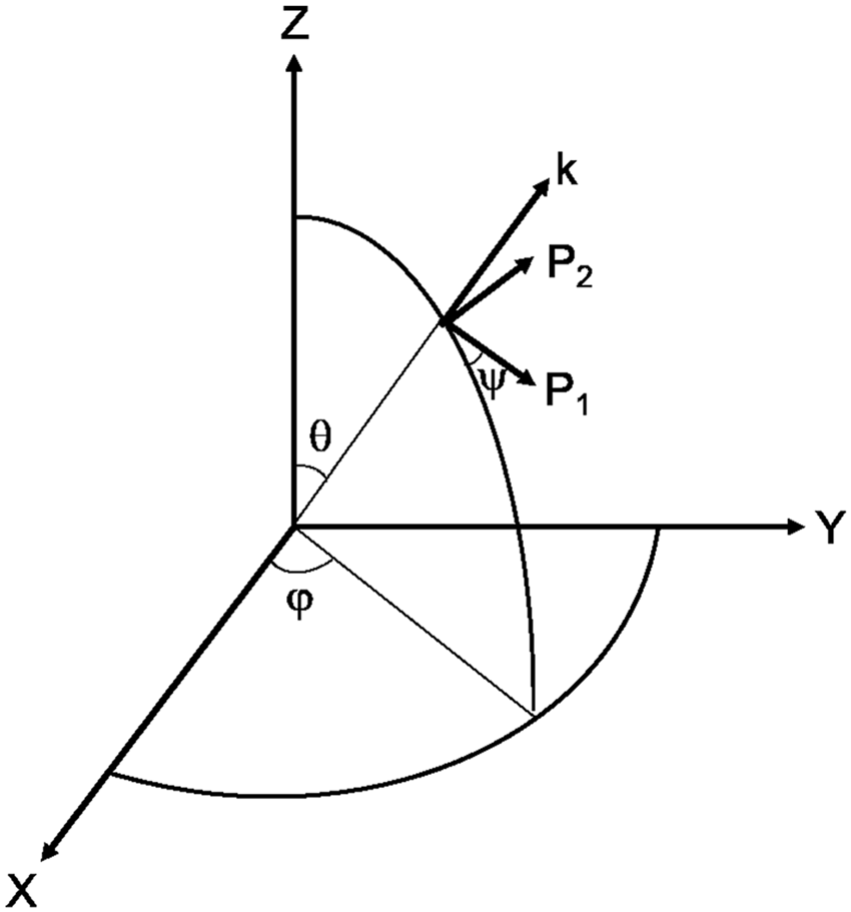
Eigen polarization direction of the wave in crystal for an electrical field applied. X, Y, and Z axes along each crystallographic axis respectively, k represents wave normal direction, P_1_ and P_2_ are eigen polarization directions.

Thus, the azimuthal angle of the eigen polarization direction can be expressed as *φ* + *ψ*, as shown in Fig. 1.

According to EO Q-switching theory, a LN crystal can obviously act as a QWP used in the pulse-on cavity by taking the value of *φ* as ±45°, for which the eigen polarization direction of the wave in the crystal is at an angle of ±45° to the transmission direction of the polarizer which is usually along the X or Y axis. The value of *θ* can be derived from equation (1) by taking the value of *δ*_1_ as π/2 + kπ (k = 0, ±1, ±2…).

As a result of *φ* =  ± 45°, equation (2) can be rewritten as

4 $${\delta }_{2}=\frac{\pi l\Delta {n}_{o}^{3}{\sin }^{2}\theta }{\lambda }\pm \frac{2\pi {n}_{o}^{3}{\gamma }_{22}El}{\lambda }$$

where “+” corresponds to *φ* = −45° and “−” corresponds to *φ* = +45°. To achieve the hold-on state, the second term in equation (4) must be π/2, which theoretically implies that the required quarter-wave voltage is equal to that of a conventional LN EO Q-switch.

The above analysis confirms that a single-block pulse-on LN EO Q-switch can be achieved using a LN crystal with a specific orientation. However, accompanying effects must be considered, including deviation in the voltage direction from the X axis, the temperature sensitivity of the natural birefringence, and the thermal expansion that is different from the one of a conventional Q-switch due to anisotropy.

According to theoretical calculations, the LN crystal is $({\rm{xztw}})-\frac{\theta }{\sqrt{2}}/\frac{\theta }{\sqrt{2}}$ cut to meet the orientation requirement. However, doing so will result in a small angular variation between the voltage direction and the X axis. The electric field E applied can be expressed as

5 $$\vec{E}={E}_{x}\vec{i}+{E}_{z}\vec{k}=E\,\cos \,\frac{\theta }{\sqrt{2}}\vec{i}-E\,\sin \,\frac{\theta }{\sqrt{2}}\vec{k}$$

According to the theory of the EO effect, the optical impermeability tensor may be written as

6 $$(\begin{array}{ccc}\frac{1}{{n}_{o}^{2}}+\gamma {}_{13}{E}_{z} & -{\gamma }_{22}{E}_{x} & {\gamma }_{51}{E}_{x}\\ -{\gamma }_{22}{E}_{x} & \frac{1}{{n}_{o}^{2}}+\gamma {}_{13}{E}_{z} & 0\\ {\gamma }_{51}{E}_{x} & 0 & \frac{1}{{n}_{e}^{2}}+{\gamma }_{33}{E}_{z}\end{array})$$

which demonstrates that *E*_z_ only influences the terms in the diagonal. Here, we consider only small values for *θ*; thus, the terms, *γ*_13_ *E*_*z*_ and *γ*_33_ *E*_*z*_, can be ignored because they are composed of the product of two small values and are much less than 1/*n*_*o*_^2^ and 1/*n*_*e*_^2^. Therefore, in our design, the effect of the deviation in the voltage direction on the index ellipsoid is negligible.

The influences of the temperature sensitivity of the natural birefringence and the anisotropic thermal expansion on the phase retardation are theoretically analyzed. According to equation (1), the variation in the phase retardation *δ*_1_ per unit change in temperature can be obtained as

7 $$\Delta {\delta }_{1}={\delta }_{1}(\frac{1}{l}\frac{\partial l}{\partial T}+\frac{2}{\Delta {n}_{o}^{3}}\frac{\partial {n}_{o}}{\partial T}-\frac{2}{\Delta {n}_{e}^{3}}\frac{\partial {n}_{e}}{\partial T}+\frac{3}{{n}_{o}}\frac{\partial {n}_{o}}{\partial T})$$

From equation (4), the variation in the phase retardation *δ*_2_ per unit change in temperature can be expressed as

8 $$\,\Delta {\delta }_{2}=\Delta {\delta }_{1}+({\delta }_{2}-{\delta }_{1})(\frac{3}{{n}_{o}}\frac{\partial {n}_{o}}{\partial T}+\frac{1}{l}\frac{\partial l}{\partial T}-\frac{1}{d}\frac{\partial d}{\partial T})\,$$

where *d* is the crystal thickness along the direction of the electric field. The values of *δ*_1_ and *δ*_2_ involved in the experiment were 5π/2 and 3π, respectively. The orders of magnitude of the thermo-optical coefficients $\frac{\partial {n}_{o}}{\partial T}$ and $\frac{\partial {n}_{e}}{\partial T}$ are approximately −5^28^, and those of the thermal expansion coefficients $\frac{1}{l}\frac{\partial l}{\partial T}$ and $\frac{1}{d}\frac{\partial d}{\partial T}$ are approximately −6^25^, which are so small that the variations in the phase retardations *δ*_1_ and *δ*_2_ with temperature can be ignored. Additionally, equation (3) shows that the angle of the eigen polarization direction is also affected by the temperature sensitivity of the natural birefringence, but the variation in Δ with temperature is sufficiently small that the change in the angle of the eigen polarization direction is negligible. Thus, the temperature sensitivity of the natural birefringence and the anisotropic thermal expansion have no influence on the temperature stability of the novel Q-switching performance.

### Experiments of the Q-switched laser

**Figure 2:**
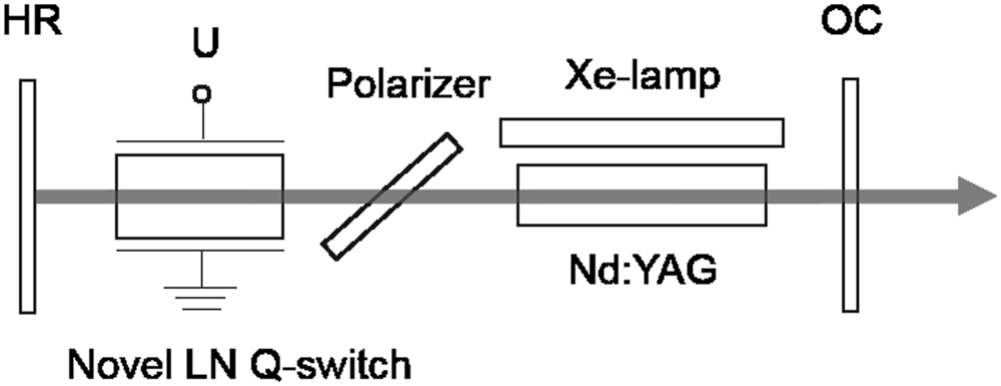
Experimental setup of the novel LN EO Q-switched Nd:YAG laser.

Based on the above analysis, we prepared a single-block pulse-on LN EO Q-switch. The LN crystal was (xztw)-1.2°/1.2°-cut with dimensions of 9 mm × 9 mm × 18.8 mm (X × Y × Z). Each face of the crystal was finely ground, the transmission surface was polished precisely and anti-reflection (AR) coated at 1064 nm. The X surface was plated with gold and chrome. The novel Q-switch was used in the Nd:YAG laser. A plano-plano cavity with a length of approximately 250 mm was employed, and the experimental setup is shown in Fig. 2. The output coupler (OC) transmission was 10%. The pump source was a Xe-lamp with an input energy of about 10 J. A Nd:YAG crystal with a doping concentration of 1.1at% and dimensions of ϕ5 mm × 80 mm was chosen as the laser crystal. The polarizer was a quartz plate oriented along the Brewster angle. A homemade EO Q-switch driver with a rise time of 7 ns and a continuously adjustable high-voltage DC power supply were used. The output energy was measured by an energy meter, and the temporal pulse behavior of the Q-switched laser was recorded using a digital oscilloscope and a fast photodiode. To confirm the practicability of the novel Q-switch, a commercial LN Pockels cell (PC), which was z-cut with dimensions of 9 mm × 9 mm × 18.8 mm (X × Y × Z), and a QWP were used in the cavity. The performance of the novel pulse-on Q-switched laser was compared with that of the conventional Q-switched laser.

The temperature dependence of the novel Q-switched laser performance was determined experimentally and compared with that of the conventional Q-switched laser. The entire laser system was placed into a high-low temperature experimental device. The experiment was conducted in the temperature range of −40 to + 65 °C with a heating rate of 2 °C/min and a cooling rate of 0.5 °C/min. The output energy at a repetition rate of 1 Hz was measured approximately every 10 °C. At each temperature test point, the temperature was equilibrated by maintaining the entire laser system at that temperature for two hours. To ensure the validity and accuracy of the test data, the experiment was repeated three times.

### Results and Discussion

The theoretical calculations determined that the proper orientation of LN at *λ* = 1064 nm are θ = 1.7° and φ = ±45°. Here, we have taken the value of *δ*_1_ to be 5π/2 and the transmission direction of the polarizer to be along the X axis; the dimensions of the LN crystal were 9 mm × 9 mm × 18.8 mm (X × Y × Z). Note that other values satisfying the requirement of *δ*_1_ = π/2 + kπ (k = 0, ±1, ±2…) could also have been chosen.

**Figure 3:**
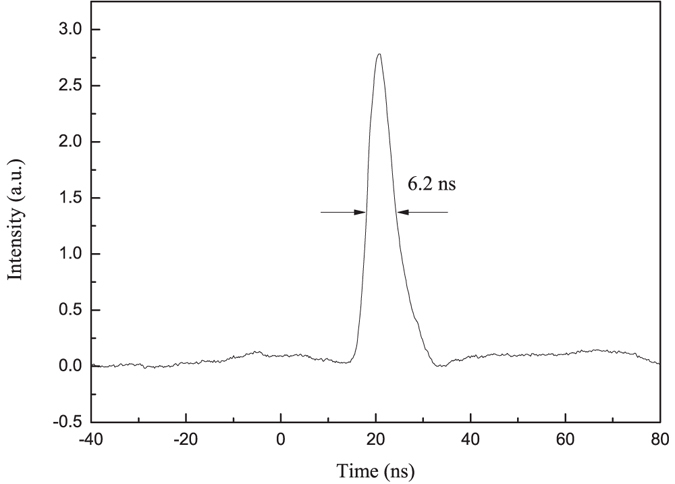
Pulse shape of the novel LN Q-switched Nd:YAG laser.

A LN crystal was (xztw)-1.2°/1.2°-cut and used in the Nd:YAG laser. We measured the following output results: the static energy E_1_ when there was only a polarizer in the cavity and the energy E_2_ when the (xztw)-1.2°/1.2°-cut crystal or the z-cut crystal and the QWP were inserted into the cavity. A continuously adjustable high DC voltage was applied to the X direction of LN, and the maximum output energy E_3_ and corresponding voltage U were recorded. When the Q-switch was in operation and the repetition rate was 10 Hz, the dynamic output energy E_4_ was measured, and the pulse shape was recorded from which the pulse width τ was obtained. All the results and the contrast ratio E_4_/E_2_ are listed in Table 1, and the pulse shape is shown in Fig. 3.

**Table 1:** Output results of the two pulse-on LN EO Q-switched Nd:YAG lasers. “—” mean that there is no energy was detected with an energy meter whose range is 0~200 μJ.

Crystal Orientation	E_1_(mJ)	E_2_(mJ)	E_3_(mJ)	E_4_(mJ)	τ(ns)	U(V)	Contrast ratio
z-cut	201	—	195	200	6.2	2200	—
(xztw)-1.2°/1.2°-cut	201	0.3	187	192	6.2	2000	640

**Figure 4:**
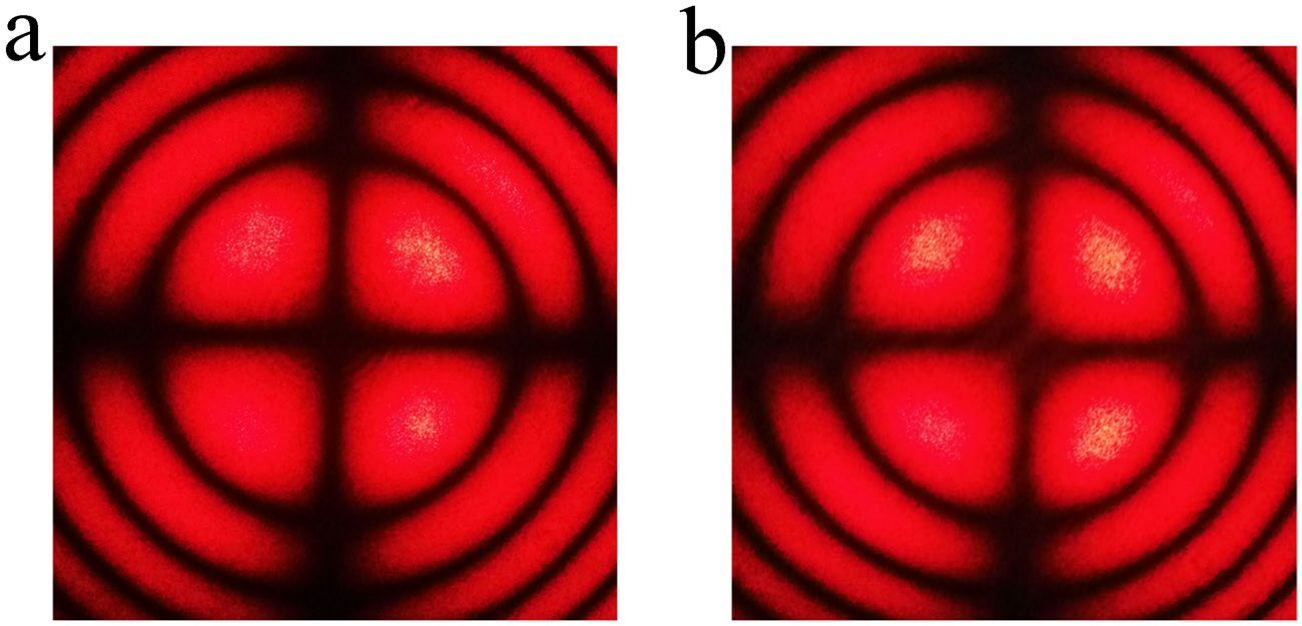
Conoscopic interference patterns for the two crystals. (**a**) z-cut LN crystal. (**b**) (xztw)-1.2°/1.2°-cut LN crystal.

Table 1 shows that the performance of the novel pulse-on Q-switched laser is slightly inferior to that of the conventional pulse-on Q-switched laser. However, the difference in the dynamic output energy E_4_ is only 4% and that of the maximum output energy E_3_ is only 4.6%, which demonstrate that the performances of the two pulse-on Q-switched lasers can be regarded as almost identical. For the novel Q-switched laser, the energy E_2_ at the hold-off state is greater than that of the conventional Q-switched laser, which mainly results in a lower contrast ratio. The main reason for these differences is thought to be because the (xztw)-1.2°/1.2°-cut crystal is subjected to stress that was generated during the optical processing and coating. These stress leads to birefringence non-uniformity and depolarization, which can easily be derived from the conoscopic interference patterns for the two LN crystals, as shown in Fig. 4. According to the theory of conoscopic interference, the slight angular variation between the optic axis and the normal of the crystal surface for the (xztw)-1.2°/1.2°-cut crystal is so small that the optical path length difference can be ignored for the light rays whose propagation directions are at the same angles to optic axis. Thus, the conoscopic interference pattern for the (xztw)-1.2°/1.2°-cut crystal should theoretically be the same as that for the z-cut crystal. However, the interference ring of the (xztw)-1.2°/1.2°-cut crystal is clearly distorted slightly, and the cross is divided, similar to that of a crystal with an excellent optical quality but acted upon by an external force, as mentioned in the literature^29^. The birefringence non-uniformity and depolarization will cause the Q-switch to incompletely hold off or on, which results in a lower stored energy and extraction efficiency and further leads to a lower output energy and peak power. Thus, the laser Q-switched by the (xztw)-1.2°/1.2°-cut crystal can be expected to achieve greater outputs once the stress is released. In addition, although a QWP is saved, the dynamic output energy of the novel Q-switched laser will not increase significantly because the loss induced by the QWP is far less than other losses in the cavity. Note that the novel Q-switch, even if its birefringence uniformity is affected by stress, can still satisfy the requirements of practical applications. Additionally, our novel Q-switch will certainly be more helpful for the compactness and stability of the cavity than a conventional pulse-on Q-switch. Table 1 also shows that the static quarter-wave voltages U of the two Q-switches are nearly equivalent within a reasonable error range, which conforms to the theoretical analysis.

**Figure 5:**
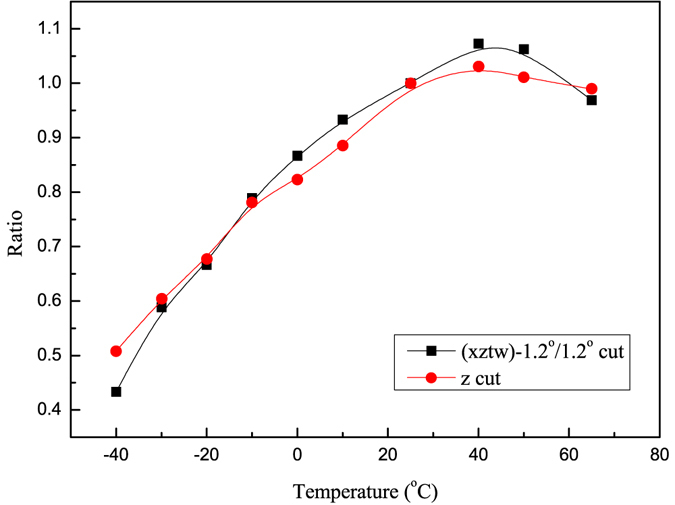
The ratios of output energy at every temperature to that at room temperature versus temperature.

The high-low temperature experiment was repeated three times, and the maximum variation in the test data is less than 3%, which demonstrates the reliability and validity of the experiment. The output energy at every temperature point was compared with the one at room temperature. The ratios of energy at every temperature to that at room temperature are found to vary with temperature, as shown in Fig. 5. A similar changing tendency is found for the novel Q-switch we designed and the conventional pulse-on Q-switch, and the maximum difference in the ratios is less than 0.07. Thus, the novel Q-switch we designed shows the same temperature dependence as the conventional one, which verifies the practicability of the novel Q-switch and further demonstrates that no additional temperature instability is caused by the temperature sensitivity of the natural birefringence and the anisotropic thermal expansion. Moreover, both Q-switches perform poorly at sub-freezing temperatures, which has been analyzed in many reports^18^. This may be caused by the pyro-electric charge buildup on the optical faces at low temperature, and it can be improved by some strategies, as mentioned in the literature^18^.

## Conclusion

We have successfully designed and prepared a single-block pulse-on LN EO Q-switch. To the best of our knowledge, this is the first time that a LN EO Q-switch operating in the pulse-on regime independently without the assistance of a QWP or analyzer has been successfully used in a Nd:YAG laser. The proper orientation of the LN crystal was determined to be θ = 1.7° and φ = ±45°, and the quarter-wave voltage was found to be identical to that of a conventional LN EO Q-switch. The analytical method is suitable for similar problems. The influence of the temperature sensitivity of the natural birefringence and the anisotropic thermal expansion on the phase retardation and the angle of the eigen polarization direction were determined to be negligible, and we verified experimentally that no additional temperature instability was caused by the temperature sensitivity of the natural birefringence and the anisotropic thermal expansion. The performance and temperature dependence of the novel Q-switched laser were found to be approximately the same as those of the conventional pulse-on EO Q-switched laser, which strongly verified the practicability of the novel Q-switch. Our novel Q-switch is highly beneficial to engineering applications, because it retains the advantages of the pulse-off regime, namely, the lower cost and the compactness and stability of the laser cavity, while also retaining the advantages of the pulse-on regime, namely, the lower requirement for the Q-switch driver and the longer lifetime.
